# A prognostic risk model based on immune‐related genes predicts overall survival of patients with hepatocellular carcinoma

**DOI:** 10.1002/hsr2.202

**Published:** 2020-11-10

**Authors:** Banglun Pan, Lin Liu, Wei Li

**Affiliations:** ^1^ Key Laboratory of Laboratory Medicine, Ministry of Education of China, School of Laboratory Medicine and Life Sciences Wenzhou Medical University Wenzhou China; ^2^ Zhejiang Provincial Key Laboratory of Medical Genetics Wenzhou Medical University Wenzhou China

**Keywords:** hepatocellular carcinoma, human leucocyte antigen‐I molecules, immune checkpoint genes, immune‐related genes, prognostic risk model

## Abstract

**Background and aims:**

Hepatocellular carcinoma (HCC) is one of the most common heterogeneous tumors that occurs after chronic liver diseases and hepatitis virus infection. Immune‐related genes (IRGs) and their ligands regulate the homeostasis of tumor microenvironment, which is essential for the treatment of HCC and its prognosis. This study aimed to investigate the clinical value of IRGs in predicting the prognosis of HCC.

**Methods:**

We downloaded RNA‐seq data and clinical information from TCGA database. Samples were randomly divided into training cohort and testing cohort. The “limma” R package was performed to identify differentially expressed IRGs (DEIRGs) between HCC group and normal group. Prognostic DEIRGs (PDEIRGs) were obtained by univariate Cox analysis. LASSO and multivariate Cox analysis were used, and a prognostic risk model was constructed. In order to better demonstrate the clinical value of our model in predicting overall survival rate, a nomogram was constructed. To further investigate the molecular mechanism of our model, gene set enrichment analysis (GSEA) was performed.

**Results:**

Compared with the low‐risk group, the high‐risk group had a significantly worse prognosis. Moreover, our prognostic risk model can accurately stratify tumor grade and TNM stage. Importantly, in our model, not only immune checkpoint genes were well predicted, but also human leucocyte antigen‐I molecules were revealed. GSEA suggested that “MAPK signaling pathway,” “mTOR signaling pathway,” “NOD like receptor signaling pathway,” “Toll like receptor signaling pathway,” “VEGF signaling pathway,” “WNT signaling pathway” had significant correlations with the high‐risk group.

**Conclusion:**

Overall, our study showed that our prognostic risk model can be used to assess prognosis of HCC, which may provide a certain basis for the survival rate of patients with HCC.

## 1 INTRODUCTION

Hepatocellular carcinoma (HCC) is caused by chronic hepatitis, cirrhosis, and liver fibrosis. The vast majority of patients, including those who exceed Milan Criteria, can only receive palliative care, with low long‐term survival.^1^ It is worth noting that the prognosis of patients with bile duct metastasis and intrahepatic hematoma is not optimistic,^2^ which may ultimately contribute to the development and treatment of HCC. Previous studies have shown that tumor‐infiltrating immune cells were highly relevant for prognosis and identification of immunotherapy targets in HCC.^3^ Therefore, identification of prognostic differentially expressed immune‐related genes (PDEIRGs) is of great significance for improving the prognosis, evaluating therapeutic effect and overall survival (OS). However, the risk assessment of IRGs in prognosis of HCC is rarely explored and further analysis is needed.

In recent years, tumor immunotherapy has received more and more attention. The success of immunotherapy strategies such as immune checkpoint (ICI) blockade in several tumors has established the role of immunotherapy.^4^ Immunotherapy can be broadly divided into ICI therapies and adoptive cell therapies (ACTs), of which ICIs mainly function through receptor/ligand recognition,^5^, ^6^ while ACTs involve the infusion of pathogen‐specific T cells from a donor to recipient.^6^ IRGs play a crucial role in regulating receptor/ligand activity in ICIs treatments.^5^ Therefore, IRGs may be used as a reference for sensitivity indexes to tumor immunotherapy and perform personalized treatment.

At present, tumor immunotherapy for HCC has achieved remarkable progress. Cytotoxic T lymphocyte‐associated antigen‐4 (CTLA‐4) and programmed death‐ligand 1 (PD‐L1) inhibitors have effectively prolonged the OS of patients with advanced HCC (including distant metastasis).^7^ However, some HCC patients were not sensitive to ICIs, which may be due to the abnormal expression of IRGs.^6^ Identification of PDEIRGs may be helpful for implement individualized treatment and evaluation of prognosis in HCC patients. In this study, we constructed a prognostic risk model based on PDEIRGs and demonstrated that our prognostic risk model has an important role in predicting the prognosis of HCC patients and contributes to individualized therapy at least to a certain extent.

## 2 METHODS

### 2.1 Sample information

RNA sequence data and clinicopathological information of HCC patients were obtained through The Cancer Genome Atlas (TCGA) database (https://cancergenome.nih.gov/), and the RNA‐seq data and clinical information were matched according to patients' ID. This study met the publication guidelines stated by TCGA. All data used in the study was obtained from TCGA, hence ethics approval and informed consent were not required.^8^ IRGs and transcription factors (TFs) terms were downloaded from the ImmPort database (https://www.immport.org/home)^9^ and Cistrome project (http://cistrome.org/),^10^ respectively.

### 2.2 Construction of the prognostic risk model

DEIRGs were identified by Wilcoxon test, and cut‐off value was set to false discovery rate (FDR) <0.05, |log2 fold‐change (FC)| >2. To improve reliability of our prognostic risk model, HCC patients (N = 370) were randomly divided into training cohort (N = 185) and testing cohort (N = 185; Table 1). PDEIRGs which used to construct our prognostic risk model were identified by LASSO and multivariate Cox analysis. The risk score was calculated by mRNA expression and estimated regression coefficients, and our prognostic risk model was validated with testing cohort and entire TCGA cohort. First, PDEIRGs were identified by univariate Cox analysis, then LASSO analysis was used to prevent the model from overfitting. Finally, multivariate Cox analysis was used to construct a prognostic risk model.

**TABLE 1:** Grouping of the HCC patients

Clinical parameters	Variable	Training cohort (N = 185)	Testing cohort (N = 185)	Entire TCGA cohort (N = 370)
Risk group	High‐risk	93 (50.27%)	92 (49.73%)	185 (50.00%)
	Low‐risk	92 (49.73%)	93 (50.27%)	185 (50.00%)
Existing status	Alive	123 (66.49%)	121 (65.41%)	244 (65.95%)
	Dead	62 (33.51%)	64 (34.59%)	126 (34.05%)

Abbreviation: HCC, hepatocellular carcinoma.

### 2.3 Risk score calculation

To calculate risk score of each HCC patient, we calculated the estimated regression coefficients by multivariate Cox analysis. Patients were divided into high/low‐risk groups based on the risk score.^11^ The following computational formula was used for this analysis:

$$\text{Risk score}=\sum_{i=1}^{n}\left(\text{coefficient of}\;\left(\text{gene}\;i\right)\times \text{expression value of}\;\left(\text{gene}\;i\right)\right)$$

gene *i* represents the *i*th gene, and coefficient of (gene *i*) represents the estimated regression coefficient of the *i*th gene.

### 2.4 Selection of immune checkpoint genes and human leucocyte antigen‐I

We also studied the relationship between human leucocyte antigen‐I (HLA‐I) molecules and our prognostic risk model. A list of 24 HLA‐I molecules were derived from TSNAdb database (http://biopharm.zju.edu.cn/tsnadb/).^12^ We investigated three genes previously reported to be crucial targets of immune checkpoint inhibitors: programmed cell death 1 ligand 1 (PD‐L1), cytotoxic T‐lymphocyte associated protein 4 (CTLA‐4), T cell immunoglobulin‐3 (TIM‐3).^13^, ^14^, ^15^

### 2.5 Kyoto Encyclopedia of genes and genomes enrichment analysis

In order to explore the potential immune molecular mechanisms and immune pathways underlying our prognostic risk model, we conducted gene set enrichment analysis (GSEA) to find enrichment items predicted to correlated with Kyoto Encyclopedia of genes and genomes (KEGG) pathways. Family‐wise error rate (FWER) *P* < .01 and FDR *q* < .01 were considered statistically significant. “c2.cp.kegg.v7.0.symbols.gmt” were applied in GSEA analysis.^16^, ^17^

### 2.6 Construction and validation of a predictive nomogram

Nomogram makes the results of our prognostic risk model more readable and has higher clinical value. All independent prognostic factors determined by multivariate Cox analysis were included to establish a nomogram to determine the possibility of three‐OS in the patients with HCC. Then we evaluated the effectiveness of the nomogram. The calibration curve of the nomogram was drawn to observe the prediction probability of the nomogram relative to the observation rate. Subsequently, we used the time‐dependent receiver characteristic operator (ROC) curve to compare the nomogram that includes all independent prognostic factors with the nomogram that includes only one.

### 2.7 Statistical analysis

R software was used to perform all statistical analyses, and *P* < .05 was considered statistically significant. Quantitative variables were analyzed using a *t* test for paired samples or Wilcoxon rank‐sum test for unpaired samples as appropriate. Person correlation coefficient test was used to determine the rank correlation among the different variables. Kaplan‐Meier analysis with Wilcoxon rank sum test/log‐rank test was used to analyze the survival outcomes between the high/low‐risk groups using the R package “Survival” and “Survminer,” Wilcoxon rank sum test was used in the training/entire TCGA cohorts and log‐rank test was used in the testing cohort. Multivariate Cox analysis was used to identify whether our prognostic risk model could be used as an independent prognostic factor for the prognosis of HCC. Time‐dependent ROC analysis was used to evaluate the accuracy of our prognostic risk model.^18^, ^19^

## 3 RESULTS

### 3.1 Expression of IRGs in HCC

**FIGURE 1:**
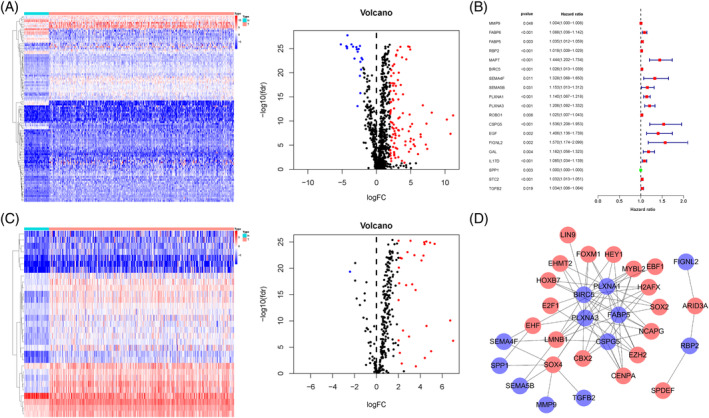
Expression profiles of immune‐related genes (IRGs), transcription factors (TFs), and TF‐based regulatory network in hepatocellular carcinoma (HCC). (A) Heatmap and volcano plot of IRGs. (B) Identification of prognostic differentially expressed IRGs (PDEIRGs) through univariate Cox analysis. (C) Heatmap and volcano plot of TFs. (D) TF‐based regulatory network. The blue dots represent PDEIRGs, the red dots represent differentially expressed TFs that correlated with PDEIRGs in terms of their mRNA expression

The clinical information of 377 HCC patients were shown in Table S1. The mRNA expression of 2498 IRGs in HCC tissues and adjacent tissues was examined. As shown in Figure 1A, compared with adjacent tissues, there were 116 DEIRGs in HCC tissues, among which mRNA of 96 genes were found to be significantly up‐regulated, while that of 20 genes were down‐regulated. In order to study the predictive value of DEIRGs in HCC, univariate Cox analysis was performed. As shown in Figure 1B, of the 116 DEIRGs, 19 genes (PDEIRGs) were significantly associated with OS of the HCC patients.

### 3.2 Construction of TFs‐regulatory network

To further explore PDEIRGs involve in regulating network, the relationship between PDEIRGs and differentially expressed TFs (DETFs) was analyzed. Compared with adjacent tissues, there were 31 DETFs in HCC tissues (Figure 1C). Then, the correlation between 31 DETFs and 19 PDEIRGs were detected (correlation coefficient >0.3 and *P* < .05), showing that there was a significant correlation between 19 DETFs and 12 PDEIRGs. Furthermore, “Cytoscape” software was performed to construct a TFs‐regulatory network to reveal a direct correlation (Figure 1D).

### 3.3 Construction of the four‐PDEIRG‐based prognostic risk model

**FIGURE 2:**
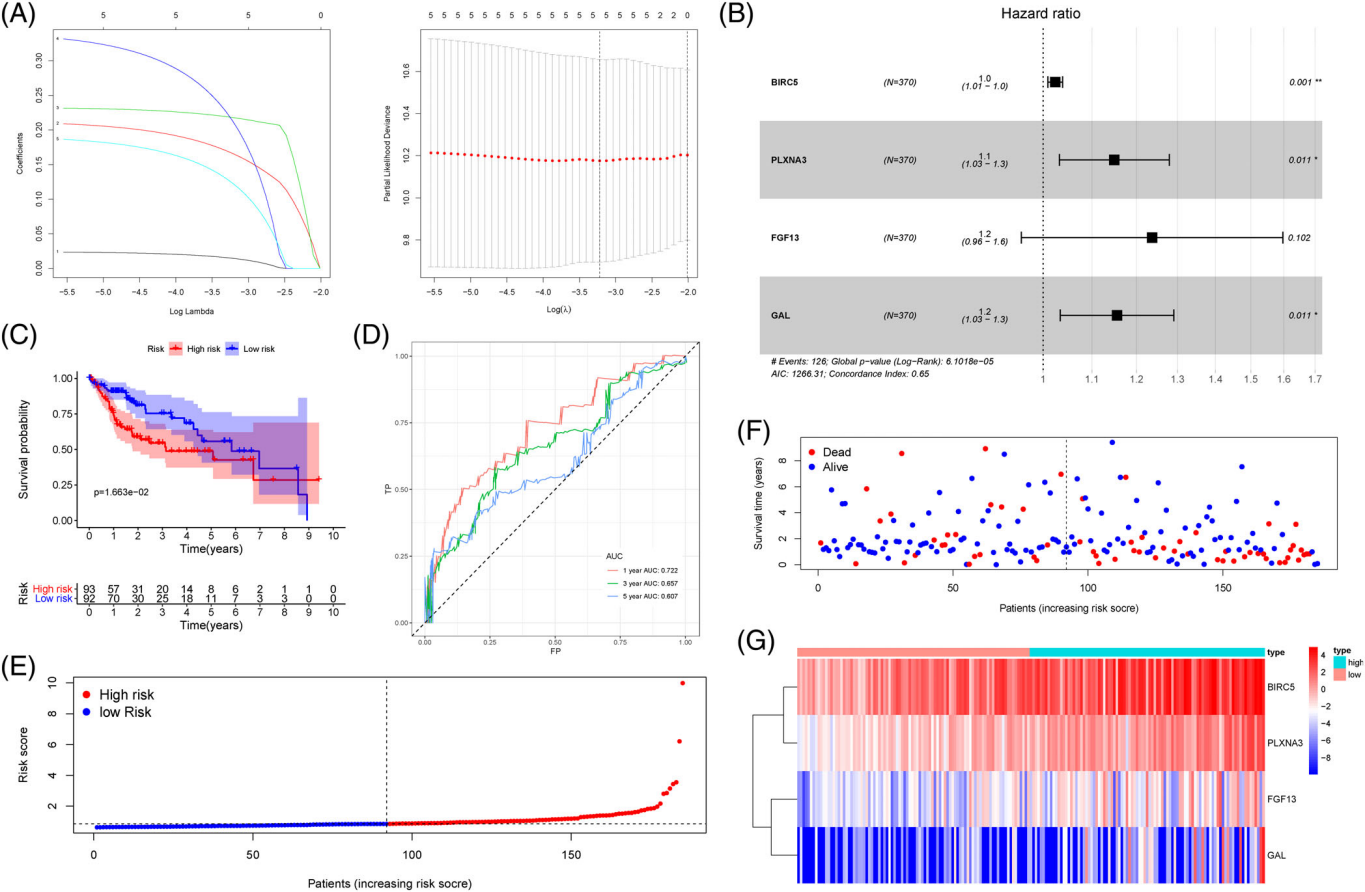
Construction of a novel prognostic risk model. (A) LASSO coefficient profiles of four prognostic differentially expressed immune‐related genes (IRGs). (B) The hazard ratio and 95% confidence interval established by multivariate Cox analysis were shown in the forest plot. Kaplan‐Meier curve. (C) Time‐dependent receiver characteristic operator curve, (D) risk curve, (E) ranked risk scores, (F) and analyzed distribution (G) in the training cohort

Among 377 HCC patients, seven of them belonged to the same samples with different order numbers, so they were excluded. A total of 370 patients were randomly separated into a training cohort (N = 185) and testing cohort (N = 185). The baseline characteristics were summarized in Table S2. In order to study the predictive value of PDEIRGs in HCC, LASSO‐modified Cox analysis was carried out in the training cohort to further narrow the scope of PDEIRGs, thereby determining the risk genes suitable for constructing the prognostic risk model (Figure 2A, Table 2). BIRC5, PLXNA3, FGF13, and GAL were selected for subsequent analysis (Figure 2B). We calculated a risk score of each HCC patient based on the mRNA expression and regression coefficients of four genes. The following computational formula was used for this analysis: Risk score = 0.024 × BIRC5 expression+0.139 × PLXNA3 expression+0.213 × FGF13 expression+0.144 × GAL expression. It is worth noting that the regression coefficient of BIRC5 is weak, but significant, indicating that even though its regression coefficient is weak, it does affect the prognosis of HCC. We then calculated the risk score for each HCC patient and used the “Survminer” R package to find the optimal cut‐off for the risk score. According to the risk scores of the patients, the patients in the training cohort were divided into the high‐ and the low‐risk group. Kaplan‐Meier curve and the time‐dependent ROC were used to evaluate the prognostic ability of our prognostic risk model. The prognosis of patients in the high‐risk group was worse than that in the low‐risk group in the training cohort (Figure 2C). The area under the ROC (AUC) values at 1, 3, 5‐year in the training cohort were 72.2%, 65.7%, and 60.7%, respectively, which showed that our prognostic risk model had good prediction ability (Figure 2D). Risk curve, ranked risk scores, and analyzed distribution in the training cohort were shown in Figures 2E–G.

**TABLE 2:** Multivariate Cox analysis of four PDEIRGs

Gene	Regression coefficients	HR	HR.95L	HR.95H	*P*‐value
BIRC5	0.024	1.024	1.009	1.039	.001
PLXNA3	0.139	1.149	1.032	1.279	.011
FGF13	0.213	1.237	0.958	1.597	.102
GAL	0.144	1.155	1.034	1.291	.011

Abbreviations: HR, hazard ratio; PDEIRGs, prognostic differentially expressed immune‐related genes.

**FIGURE 3:**
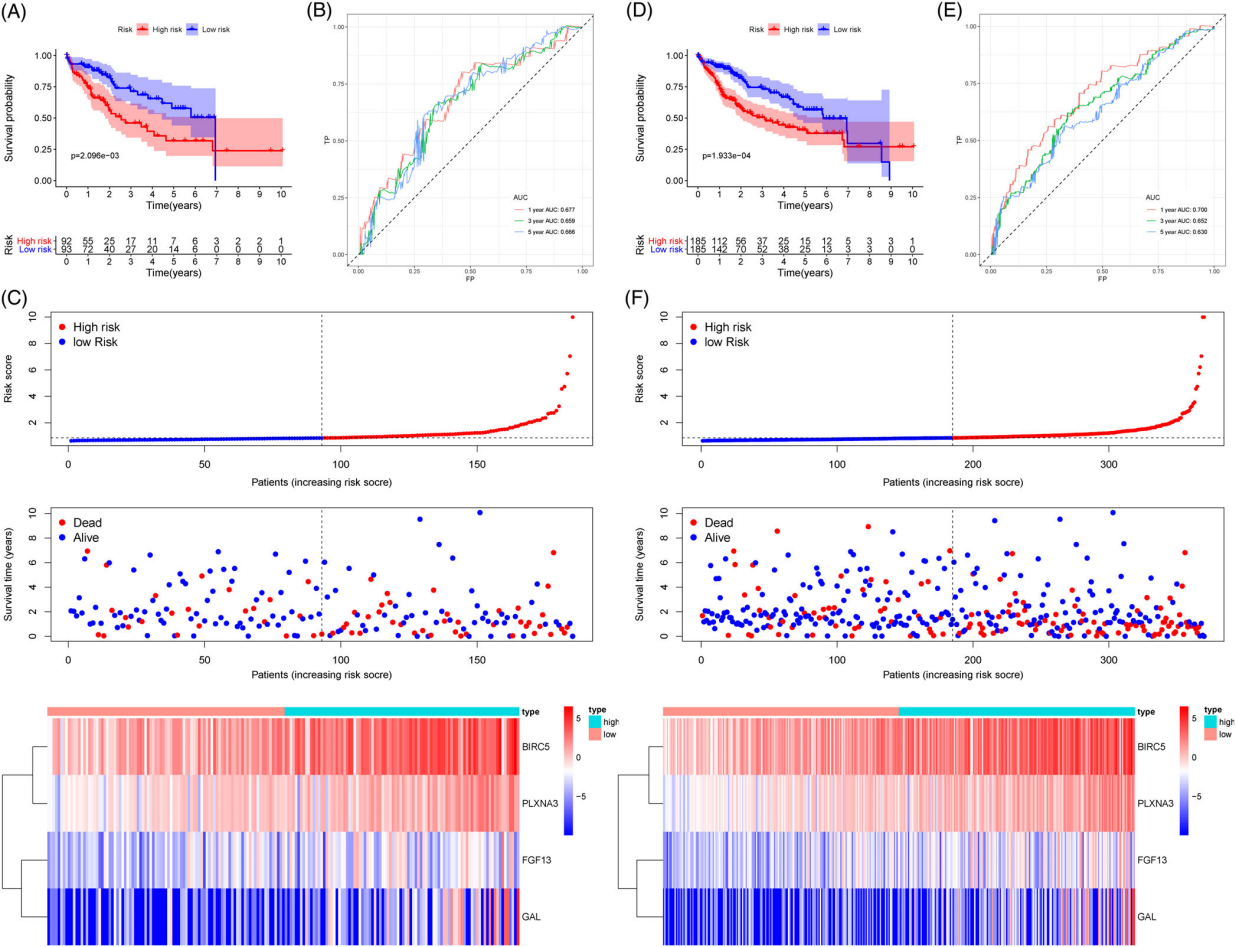
Validation of our prognostic risk model. Kaplan‐Meier curve (A), time‐dependent receiver characteristic operator (ROC) curve (B), risk score charts (C) in the testing cohort. Kaplan‐Meier curve (D), time‐dependent ROC curve (E), risk score charts (F) in entire TCGA cohort

Using the optimal cut‐off calculated from the training cohort, we divided the testing cohort and entire TCGA cohort into two risk groups. In two different cohorts, the OS was lower in the high‐risk group than which in the low‐risk group (Figure 3A,D). AUC values at 1‐, 3‐, 5‐year were 67.7%, 65.9%, and 66.6% in the testing cohort, while 70.0%, 65.2%, 63.0% in entire TCGA cohort (Figure 3B,E). To study patients' risk in two cohorts, we plotted risk curves, ranked risk scores, and analyzed distribution (Figure 3C,F).

### 3.4 Evaluation of independent prognostic value of our prognostic risk model

**FIGURE 4:**
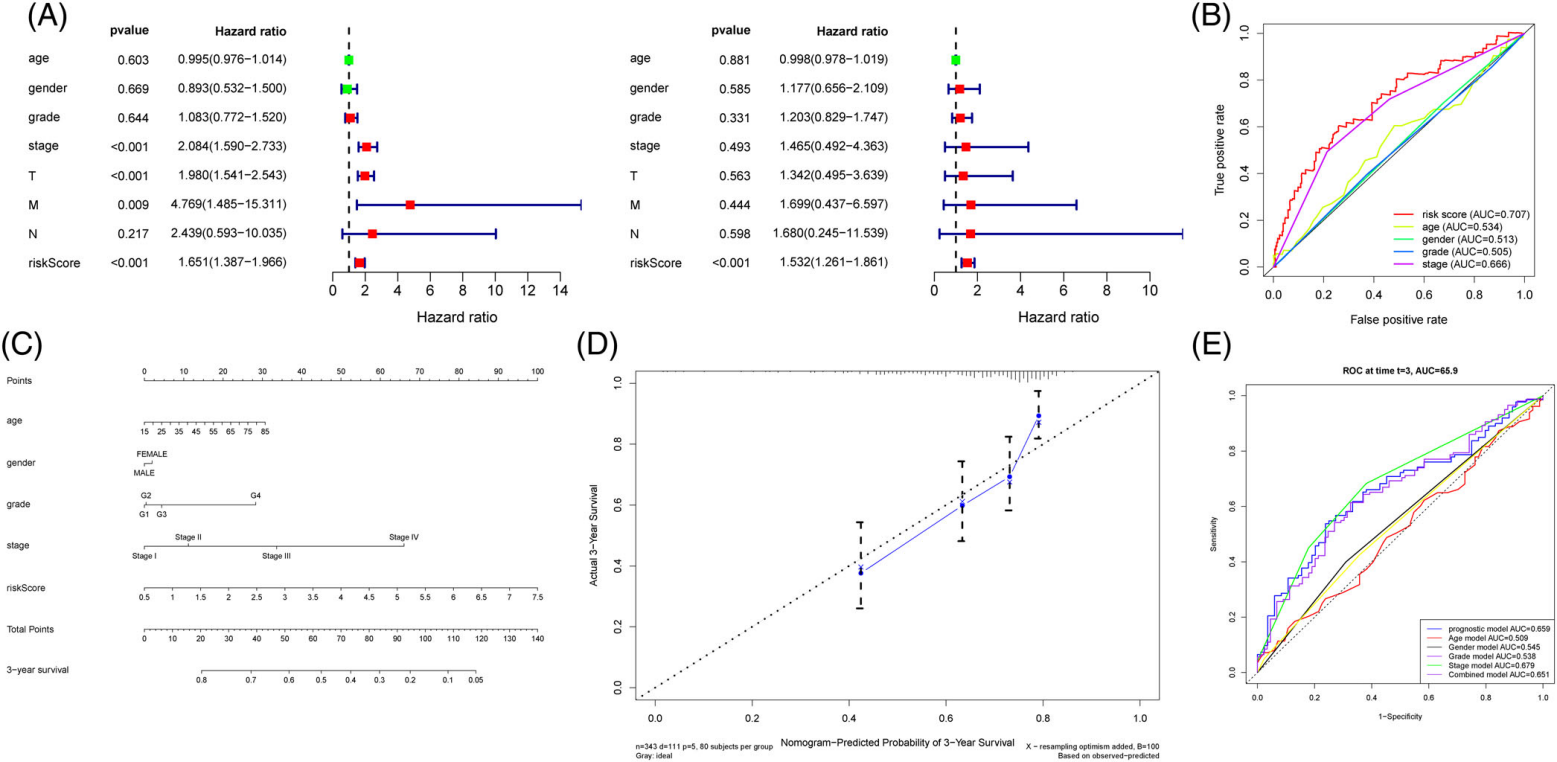
Characteristics of our prognostic risk model in entire TCGA cohort. A, Univariate and multivariate Cox analysis of our prognostic risk model. B, Time‐dependent ROC curve analysis of our prognostic risk model and other clinical characteristics at 3‐year. C, For each patient, five lines were drawn upward to determine the points received from the five predictors in the nomogram. The sum of these points was located on the “Total Points” axis. Then a line was drawn downward to determine the possibility of 3‐year overall survival of hepatocellular carcinoma. D, The calibration plot for internal validation of the nomogram. The Y‐axis represented actual survival, and the X‐axis represented nomogram‐predicted survival. E, The time‐dependent ROC curves of the nomogram compared for 3‐year overall survival in hepatocellular carcinoma

In order to determine whether our prognostic risk model could be used as an independent predictive factor, we used univariate and multivariate Cox analysis. Cox analysis showed that risk score calculated from our prognostic risk model was associated with the patients' OS (Figure 4A). To evaluate the accuracy of the risk score in predicting the survival status of HCC patients, the ROC curve of clinical parameters was plotted (Figure 4B). These results indicated that risk score can accurately reveal prognosis and may be more accurate than other clinical parameters.

### 3.5 Construction and validation of a predictive nomogram

We then used five independent prognostic factors including age, gender, TNM stage, tumor grade, and risk score to establish a nomogram to predict the 3‐year OS of HCC patients (Figure 4C). As shown in Figure 4D, the calibration plot showed that the nomogram (combined model) could accurately estimate mortality. The AUCs of our prognostic risk model, age model, gender model, tumor grade model, TNM stage model, and combined model were 0.659 (95% confidence interval [CI] 0.576‐0.742), 0.509 (95% CI 0.421‐0.598), 0.545 (95% CI 0.472‐0.618), 0.538 (95% CI 0.459‐0.617), 0.679 (95% CI 0.605‐0.754), 0.651 (95% CI 0.602‐0.787) for 3‐year OS, respectively (Figure 4E).

### 3.6 Association between our prognostic risk model and clinical characteristics of HCC

**FIGURE 5:**
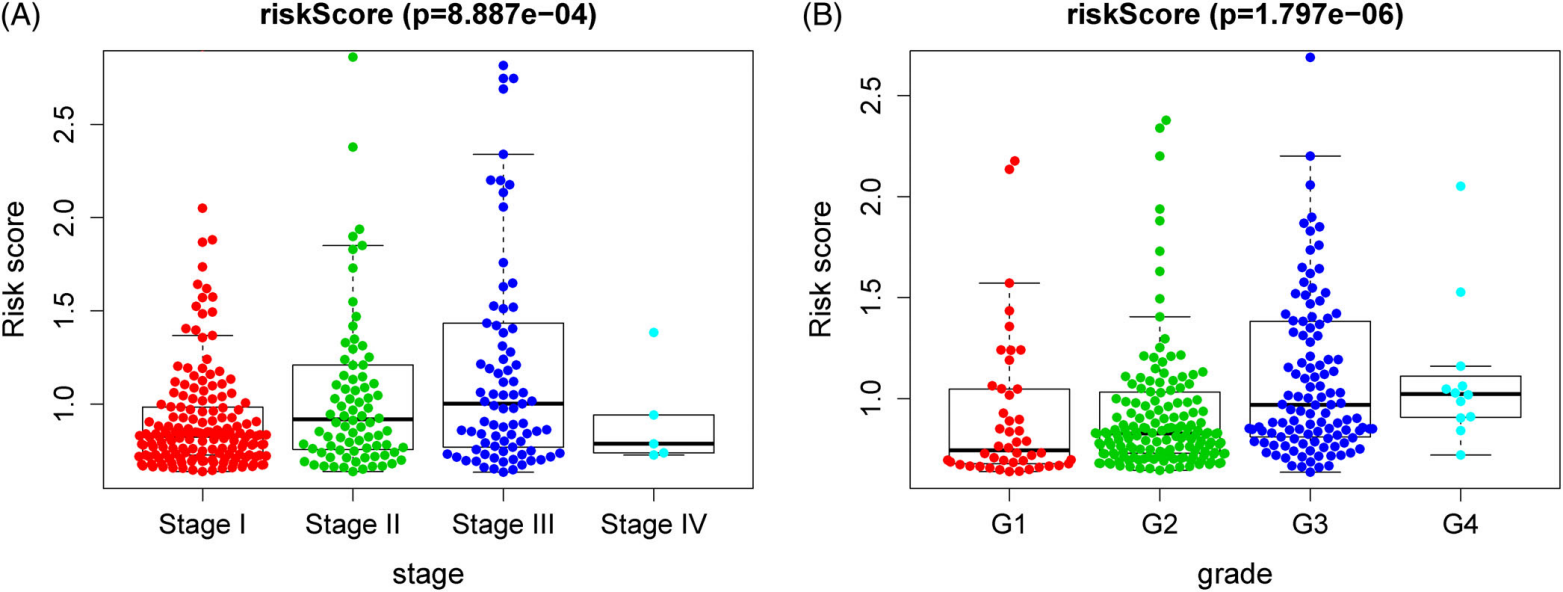
The different risk scores in hepatocellular carcinoma. The risk scores were group by TNM stage (A) and tumor grade (B)

In order to evaluate the role of our prognostic risk model in predicting the tumor biological behavior of HCC, Pearson correlation analysis was used to analyze the relationship between risk score and clinical information including age, gender, TNM stage, tumor grade. As shown in Figure 5, we found that there were different risk scores in the groups by tumor grade, TNM stage, which implied that the capacity of invasion and metastasis of high‐score HCC samples was significantly higher.

### 3.7 Correlation between our prognostic risk model and immune genes expression

**FIGURE 6:**
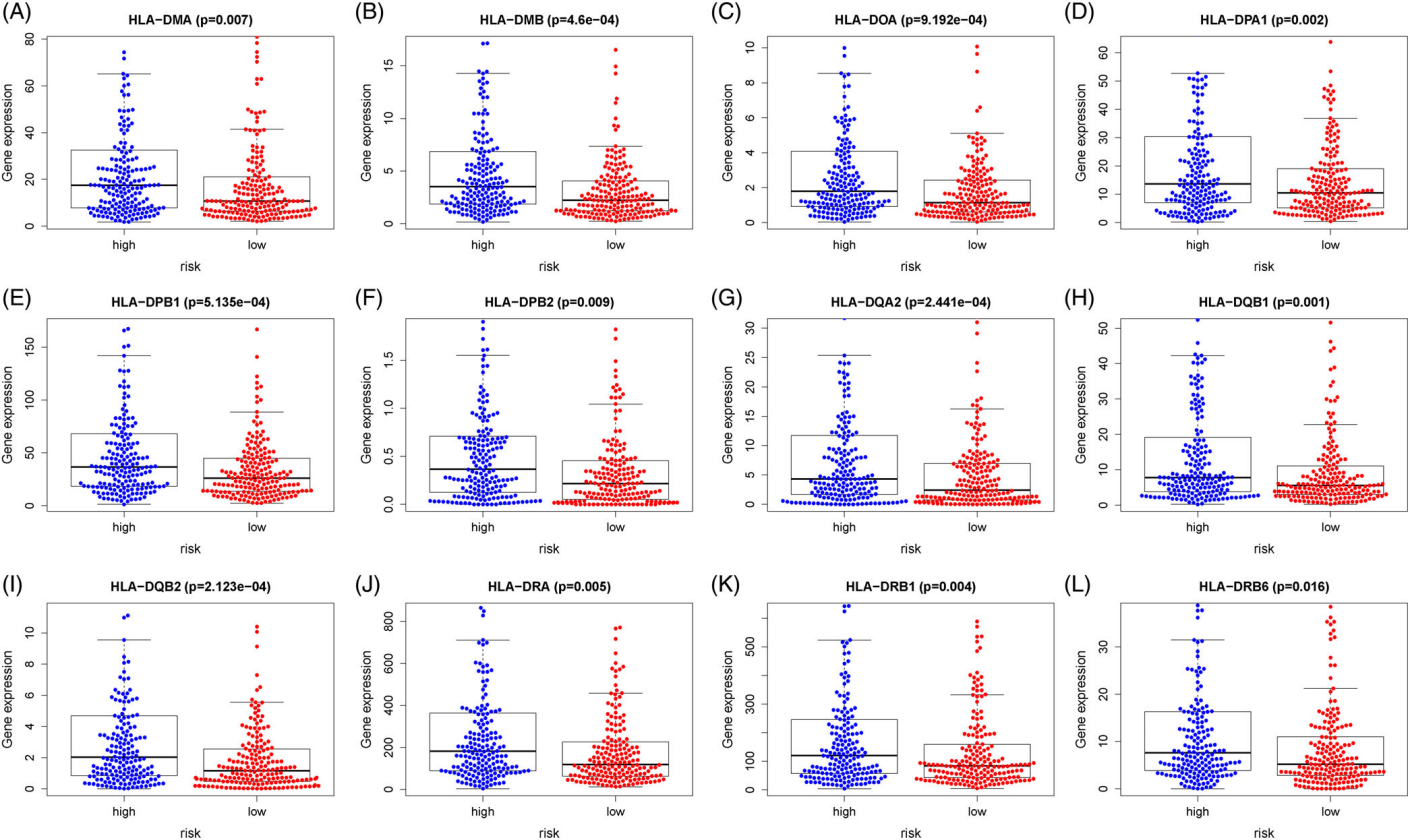
Relationship between our prognostic risk model and HLA‐I expression in entire TCGA cohort. Risk score and HLA‐DMA (A), HLA‐DMB (B), HLA‐DOA (C), HLA‐DPA1 (D), HLA‐DPB1 (E), HLA‐DPB2 (F), HLA‐DQA2 (G), HLA‐DQB1 (H), HLA‐DQB2 (I), HLA‐DRA (J), HLA‐DRB1 (K), HLA‐DRB6 (L)

In the process of new antigen presentation and T cell lysis, the key step is controlled by HLA‐I, which presents intracellular polypeptides on the cells surface for T cell receptor recognition. Down‐regulation of HLA‐I may reduce antigen presentation and promote immune escape, which is prevalent in a series of cancers and is associated with poor prognosis.^20^, ^21^ As shown in Figure 6, compared with the low‐risk group, HLA‐DMA, HLA‐DMB, HLA‐DOA, HLA‐DPA1, HLA‐DPB1, HLA‐DPB2, HLA‐DQA2, HLA‐DQB1, HLA‐DQB2, HLA‐DRA, HLA‐DRB1, and HLA‐DRB6 were higher in the high‐risk group.

**FIGURE 7:**
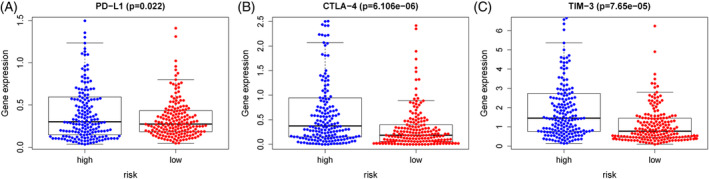
Association between crucial immune checkpoint genes and our prognostic risk model. Significant positive association between our prognostic risk model and PD‐L1 (A), CTLA‐4 (B), and TIM‐3 (C)

Tumor escape from the surveillance of immune system by multiple ways, in which controlling access of ICIs is an important process of tumor immune escape.^22^ At present, CTLA‐4 and PD‐L1 are the two important routes for tumor immune escape. The mechanism of tumor treatment by immunosuppression is to inhibit the activation of ICI pathways and avoid T cell inactivation, so as to enhance the anti‐tumor immune activity.^23^ In our study, we evaluated three key ICIs: PD‐L1, CTLA‐4, and TIM‐3. We found that our prognostic risk model was positively related to them, suggesting that our model may be used for evaluation and measurement of response to ICIs in HCC (Figure 7).

### 3.8 Prognostic risk model mediated multiple immune‐related pathways

**FIGURE 8:**
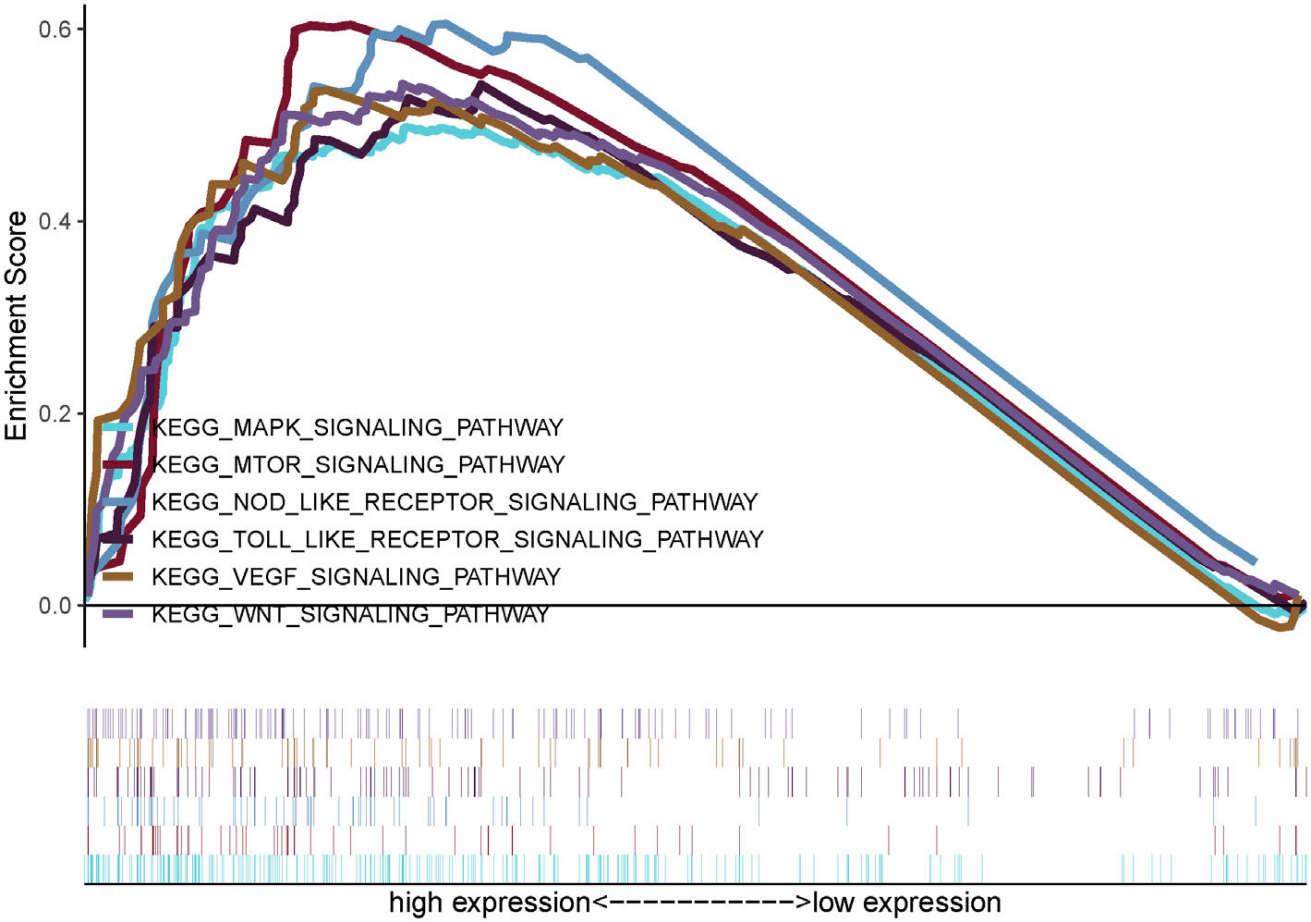
Several immune‐related pathways were up‐regulated via our prognostic risk model. A, These immune‐related pathways include MAPK signaling pathway, mTOR signaling pathway, NOD like receptor signaling pathway, Toll like receptor signaling pathway, VEGF signaling pathway, WNT signaling pathway

In order to explore the underlying molecular mechanisms and the signaling pathways of our prognostic risk model, we performed GSEA to compare the high‐risk group and the low‐risk group in HCC. KEGG enrichment suggested that “MAPK signaling pathway,” “mTOR signaling pathway,” “NOD like receptor signaling pathway,” “Toll like receptor signaling pathway,” “VEGF signaling pathway,” “WNT signaling pathway” had significant correlations with the high‐risk group (Figure 8A).

## 4 DISCUSSION

ICIs, such as PD‐L1, play an important role in the treatment of HCC.^24^ However, some patients are not sensitive to ICIs, and even worsen after treatment. The OS rate of immune‐related adverse events, including hepatic, is dramatically improved in patients treated with a combination of CTLA‐4 and PD‐L1 inhibition.^25^, ^26^, ^27^ For example, lung cancer patients treated with either pembrolizumab or nivolumab, the median time to onset of anti‐PD‐1 and anti‐PD‐L1 therapy‐induced hepatotoxicity was 25 weeks (range: 4‐31 weeks), while it was 4 weeks (range: 0.1‐23 weeks) in the melanoma patients treated with nivolumab, and 19 weeks (range: 0.3‐93 weeks) in those treated with pembrolizumab.^28^ The appearances may be due to the disorder in the binding of ICs to ICIs, in addition, the differential expression of IRGs directly affects the sensitivity and affinity of this binding.^29^ Therefore, it is important to identify PDEIRGs and determine their roles in the sensitivity of HCC patients to the efficacy of ICIs. In our study, we analyzed expression of 2498 IRGs in HCC patients and found 116 DEIRGs, of which 96 were up‐regulated and 20 down‐regulated. Using IRGs to enhance immune response and destroy immune escape state of tumor cells has become one of the important strategies to tumor immunotherapy. Some works have shown that local application of IL‐12 can better exert antitumor effects. On the one hand, IL‐12 directly involve in tumor characteristics in an autocrine manner, on the other hand, IL‐12 is effectively expressed locally in the tumor cells, activating immune cells in the form of paracrine.^30^

Constructing a prognostic risk model based on immune cells or IRGs to predict prognosis has been reported. Chen et al constructed a model based on 22 immune cells to evaluate the prognosis of patients with HBV‐related HCC.^31^ To evaluate the clinical characteristics and prognosis of HCC patients, a prognostic risk model was constructed by collecting five different HCC databases.^32^ These results suggested that the evaluation of the changes in immune cells or immune genes is vital for the prognosis of HCC. In this research, a novel prognostic risk model was constructed to predict prognosis and immunotherapy effect in HCC. Our prognostic risk model not only accurately reflected OS of HCC, but also proved risk score can be used as an independent factor to evaluate prognosis. More importantly, our prognostic risk model can effectively reflect the TNM stage and tumor grade, and help to implement personalized treatment.

HLA‐I plays a key role in process of antigen presentation and killer lymphocytes (CTLs) recognition of tumor cells. The reduction or absence of HLA‐I expression is generally considered to be the mechanism by which tumor cells escape CTL cell killing. Seventy sections of HCC patients (56 blacks, 14 Caucasians) were used for tissue staining, which proving that HLA‐I expression was up‐regulated in 94.3% of cases.^33^ Furthermore, HLA‐I is expressed in at least 10 human hepatoma cell lines,^34^ indicating the HLA‐I may regulate tumor biological process. We found that our prognostic risk model was inversely related to various HLA‐I expression. The ICI pathways consisting of PD‐1/CD279 and related ligand PD‐L1/CD274 evades immune surveillance during T cell‐mediated immune killing. Extensive evidence suggested that blocking PD‐1/PD‐L1 interactions can enhance immune normalization and anti‐cancer responses.^35^, ^36^ Among patients receiving sorafenib, the objective response rate was 55%, suggesting that only a partial benefit, although OS was extended by 15.6 months. In addition, PD‐1/PD‐L1 blockers have lower liver toxicity than conventional drugs.^25^ In our study, PD‐L1, CTLA‐4, and TIM‐3 were observed to be positive with risk score, which was consistent with previously reports.

Although our study also constructed a prognostic risk model based on tumor immune microenvironment of HCC, it differed from previous studies. First, we predicted PDEIRGs through Cox and LASSO analysis, which effectively avoids overfitting, and these genes have not been reported in previous prognostic risk models. Second, our study established a novel four‐IRG prognostic risk model and a nomogram to predict the OS of HCC, which may help individual clinical decision making for treatment. Third, we verified that our prognostic risk model can accurately reflect the clinical stratification and prognosis of HCC. Since our prognostic risk model reduces the need for whole‐genome sequencing for all HCC patients, it may be more routine and cost‐effective in practice. Fourth, the calibration chart shows that the nomogram (our prognostic risk model and combined model) can more accurately assess mortality. More importantly, our prognostic risk model shows significantly improved performance, especially in predicting the expression levels of ICIs and HLA‐I molecules, indicating that it more accurately reflects the changes in the immune microenvironment of HCC. However, in our study, there are a few shortcomings, because we only collected data from TCGA without other clinical samples. In addition, the expression and prognostic effects of these four PDEIRGs at the RNA and protein level are worthy of further study, all the mechanical analyses in our study are descriptive, and further functional experiments are needed to clarify the underlying mechanisms of them. In sum, based on the above results, we believed that our prognostic risk model can accurately reflect the clinical stratification of HCC, and predict OS of patients with different risks, which was helpful for the risk assessment of prognosis.

## FUNDING

This work is supported by the National Natural Science Foundation of China (81970753). The funder played no role in study design; collection, analysis, and interpretation of data; writing of the report; or the decision to submit the report for publication.

## CONFLICT OF INTEREST

The authors declare there is no conflict.

## AUTHOR CONTRIBUTIONS

Conceptualization: Lin Liu, Banglun Pan

Formal Analysis: Banglun Pan

Funding Acquisition: Wei Li

Writing ‐ original draft: Banglun Pan

Writing ‐ review and editing: Lin Liu, Wei Li

 All authors have read and approved the final version of the manuscript.

 Wei Li had full access to all of the data in this study and takes complete responsibility for the integrity of the data and the accuracy of the data analysis.

## TRANSPARENCY STATEMENT

Wei Li affirms that this manuscript an honest, accurate, and transparent account of the study being reported; that no important aspects of the study have been omitted; and that any discrepancies from the study as planned (and, if relevant, registered) have been explained.

## Supporting information


**TABLE S1** Clinical information of 377 HCC patients in TCGA set
**TABLE S2** Clinical features of HCC patients in training set, testing set, and entire TCGA set.

## Data Availability

The authors confirm that the data supporting the findings of this study are available within the article and its supplementary materials.
