# Supplementary material for: A prognostic risk model based on immune‐related genes predicts overall survival of patients with hepatocellular carcinoma
Source: Health Sci Rep. 2020 Nov 10;3(4):e202. doi: 10.1002/hsr2.202 (PMC7654629; doi:10.1002/hsr2.202)
Supplement: Supplementary file 1 — TABLE S1 Clinical information of 377 HCC patients in TCGA set TABLE S2 Clinical features of HCC patients in training set, testing set, and entire TCGA set. [file HSR2-3-e202-s001.docx]

**TABLE S1** Clinical information of 377 HCC patients in TCGA set

| Clinical features | Number | (%) |
| --- | --- | --- |
| Existing status |  |  |
| Alive | 249 | 66.05 |
| Dead | 128 | 33.95 |
| Age |  |  |
| <=60 | 180 | 47.75 |
| >60 | 196 | 51.99 |
| unknow | 1 | 0.27 |
| Gender |  |  |
| Female | 122 | 32.36 |
| Male | 255 | 67.64 |
| Tumor grade |  |  |
| G1-2 | 235 | 62.33 |
| G3-4 | 137 | 36.34 |
| unknow | 5 | 1.33 |
| TNM stage |  |  |
| Stage I-II | 262 | 69.5 |
| Stage III-IV | 91 | 24.14 |
| unknow | 24 | 6.37 |
| T classification |  |  |
| T1-2 | 280 | 74.27 |
| T3-4 | 94 | 24.93 |
| TX | 1 | 0.27 |
| unknow | 2 | 0.53 |
| M classification |  |  |
| M0 | 272 | 72.15 |
| M1 | 4 | 1.06 |
| MX | 101 | 26.79 |
| N classification |  |  |
| N0 | 257 | 68.17 |
| N1 | 4 | 1.06 |
| NX | 115 | 30.5 |
| unknow | 1 | 0.27 |

Note. HCC: hepatocellular carcinoma, TCGA: The Cancer Genome Atlas.

**TABLE S2** Clinical features of HCC patients in training set, testing set, and entire TCGA set.

| Clinical features | Entire TCGA set | Testing set | Training set | *P*-value |
| --- | --- | --- | --- | --- |
| Total | 370 | 185 | 185 |  |
| Dead (%) | 126(34.05） | 64(34.59) | 62(33.51) | 0.8586 |
| Median age (years) | 61 | 62 | 60 | 0.9837 |
| Female (%) | 121(32.70) | 68(36.76) | 53(28.65) | 0.1727 |
| Tumor grade (%) |  |  |  | 0.5826 |
| G1-2 | 232(62.70) | 125(67.57) | 107(57.84) |  |
| G3-4 | 133(35.95) | 59(31.89) | 74(40.00) |  |
| unknow | 5(1.35) | 1(0.54) | 4(2.16) |  |
| TNM stage (%) |  |  |  | 0.683 |
| Stage I-II | 256(69.19) | 123(66.49) | 133(71.90) |  |
| Stage III-IV | 90(24.32) | 47(25.40) | 43(23.24) |  |
| unknow | 24(6.49) | 15(8.11) | 9(4.86) |  |
| T classification (%) |  |  |  | 0.847 |
| T1-2 | 274(74.05) | 136(73.51) | 138(74.60) |  |
| T3-4 | 93(25.14) | 47(25.41) | 46(24.86) |  |
| TX | 1(0.27) | 1(0.54) | 0(0.00) |  |
| unknow | 2(0.54) | 1(0.54) | 1(0.54) |  |
| N classification (%) |  |  |  | 0.725 |
| N0 | 252(68.11) | 120(64.86) | 132(71.35) |  |
| N1 | 4(1.08) | 3(1.62) | 1(0.54) |  |
| NX | 113(30.54) | 62(33.52) | 51(27.57) |  |
| unknow | 1(0.27) | 0(0) | 1(0.54) |  |
| M classification (%) |  |  |  |  |
| M0 | 266(71.89) | 131(70.81) | 135(72.97) | 0.8407 |
| M1 | 4(1.08) | 1(0.54) | 3(1.62) |  |
| MX | 100(27.03) | 53(28.65) | 47(25.41) |  |

Note. HCC: hepatocellular carcinoma, TCGA: The Cancer Genome Atlas.
